# Anthropometric study of the hip joint in Northeastern region population with computed tomography scan

**DOI:** 10.4103/0019-5413.39572

**Published:** 2008

**Authors:** KC Saikia, SK Bhuyan, R Rongphar

**Affiliations:** Department of Orthopedics, Guwahati Medical and Hospital, Guwahati - 781 032, Assam, India

**Keywords:** Anthropometric study, CT scan, anthropometric study of the hip joint, northeastern region of India

## Abstract

**Background:**

Anthropometric study of the hip joint has important clinical implications and is largely unknown for the northeastern region of India. The purpose of this study is to determine the anatomic variation of the normal hip joint among the people of the northeastern region and to statistically compare them with the available data worldwide.

**Materials and Methods:**

We evaluated 104 individuals with normal hip joints and of different ethnic backgrounds (Caucasoid and Mongoloids) clinically and by plain x- ray. One topogram of the hip joint, one axial section of the femoral head and femoral condyles of the individual was taken on CT scan. Twelve cases had center edge angle (CE) angle less than 20° (unilateral/bilateral), were considered to be dysplastic and were excluded from the study. Thus the present study includes 92 individuals (184 normal hips, Mongoloids = 45; Caucasoid = 47) between 20-70 years of age. We calculated the mean of the CE angle, acetabular angle, neck shaft angle, acetabular version, femoral neck anteversion, acetabular depth and joint space width in both sexes.

**Results:**

The mean parameters observed were as follows: acetabular angle 39.2°, centre edge angle 32.7°, neck shaft angle 139.5°, acetabular version 18.2°, femoral neck anteversion 20.4°, acetabular depth 2.5 cm and joint space width 4.5 mm.

**Conclusion:**

The parameter and its values in our series shows differences when compared to the other western literatures. The neck shaft angle and the femoral neck anteversion in our individuals was 5-6° more than the western literature. The remaining parameters were less or equal to the western literature.

## INTRODUCTION

Anthropometeric study of the hip joint has important clinical implications. A through knowledge of hip joint anatomy is a prerequisite to understand its biomechanics. Relatively little has been written on what could be considered normal in an X-ray of hip and what is considered pathological. Mean values are of no help in individual cases. It should be known how far normal standards deviate and where pathological values can be expected.^1^ More information is needed on the computed tomographic measurement of the hip joint, including its shape, its width at precise locations and influence of age, sex and congenital morphology. These normal values are needed to set the limits of significant early radiographic alteration in patients with osteoarthritis.^2^

Different authors have suggested that difference in parameters of bone exists among different races and have tried to figure out the relationship of these variations to increased development of hip osteoarthritis, femoral neck fracture and slipped capital femoral epiphysis.^2^–^5^ Wiberg pointed out the relationship of acetabular dysplasia to early development of osteoarthritis.^6^ The normal values of our population of the northeastern (NE) region are largely unknown.

The purpose of this study is to determine the anatomic variation of the normal hip joint, with computed tomographic measurement, among the people of the northeastern region and to statistically compare them with the data available world wide.

## MATERIALS AND METHODS

We evaluated 104 individuals with radiologically normal hip aged between 20 and 70 years. There were 63 males (26-70 years) and 41 females (20-66 years). The individuals of different ethnic backgrounds belonging to the northeastern region in whom CT scan of hip was done for unrelated problems and who consented to participate in the study were included. Most of the tribes of the northeast belong to the Mongoloids, characterized by wide and short face, projecting cheek bones, low broad nose and they are short in stature. The caste group Caucasoids are characterized by long head, high forehead, narrow face, long and narrow nose and they are tall in stature than the Mongoloids. Skeletally immature individuals, postoperative patients, persons not originally of the northeastern region and uncooperative individuals were excluded from the study. The individuals were evaluated clinically and by plain X-rays to rule out hip pathology. The height of each individual was measured.

We measured seven parameters of the hips with CT scan (Siemens Somatome AR star) in 104 subjects (208 hips) maintaining a fixed and specified technical configuration while taking the CT cuts. The CT scan was done in supine position with hip and knee fully extended and the lower limbs secured to the table with straps. The foot was stabilized with a specially designed wooden frame while taking the cuts. The limbs were kept in identical position and were parallel to the CT machine. The thickness of each CT cut was 5 mm.

Twelve individuals in our study had center edge (CE) angle of less than 20° (unilateral = 10/bilateral = 2), were considered to be dysplastic and hence excluded from the study. Thus the present study includes 92 individuals (184 normal hips, Mongoloids = 45; Caucasoid = 47).

Three axial sections, two proximal and one distal were taken as described by Murphy *et al*. with CT scan.^7^ One image defines the center of the femoral head, the second image defines the base of the femoral neck and the third image defines the distal femoral condylar axis. One topogram of the hip joint up to the mid-thigh was also taken. All measurements were taken on CT scan. Geometrical reconstruction was made where necessary, e.g. femoral neck anteversion. Transparent scale and protector was used in measuring the parameters on the CT scan films.

Acetabular anteversion was measured on axial section that passed through the center of the hip joint by computed tomography which corresponds to the anatomical anteversion described by Murray.^8^

**Figure 1:**
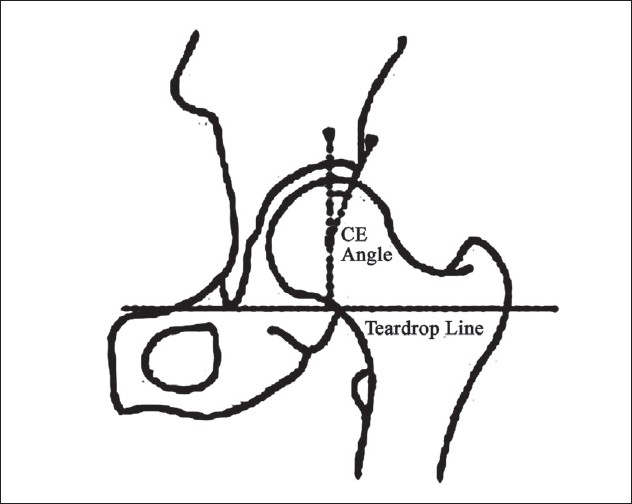
Line diagram showing CE angle of Wiberg

**Figure 2:**
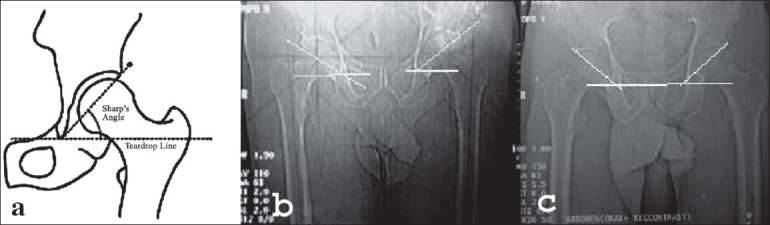
Line diagram and CT scan of acetabular angle showing increased acetabular angle (48 degree) (b) and decreased acetabular angle (30 degree) (c)

**Figure 3:**
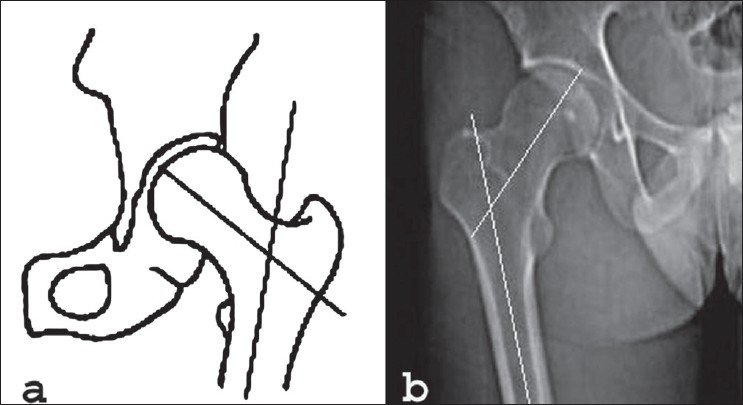
Line diagram (a) and CT scan (b) of neck shaft angle showing increased neck shaft angle (148 degree)

**Figure 4:**
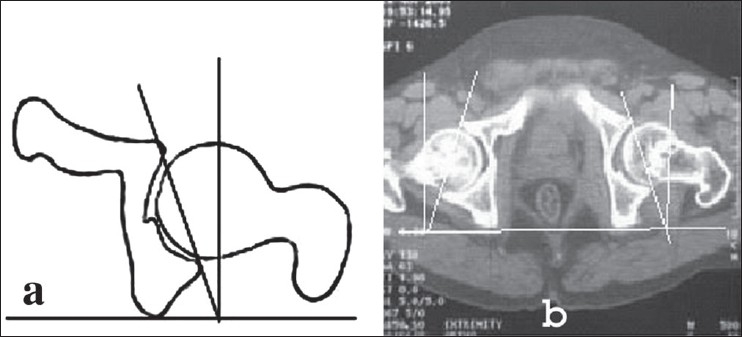
Line diagrm and (a) CT scan (b) of acetabular version showing increased acetabular version on left side -20 degree

**Figure 5:**
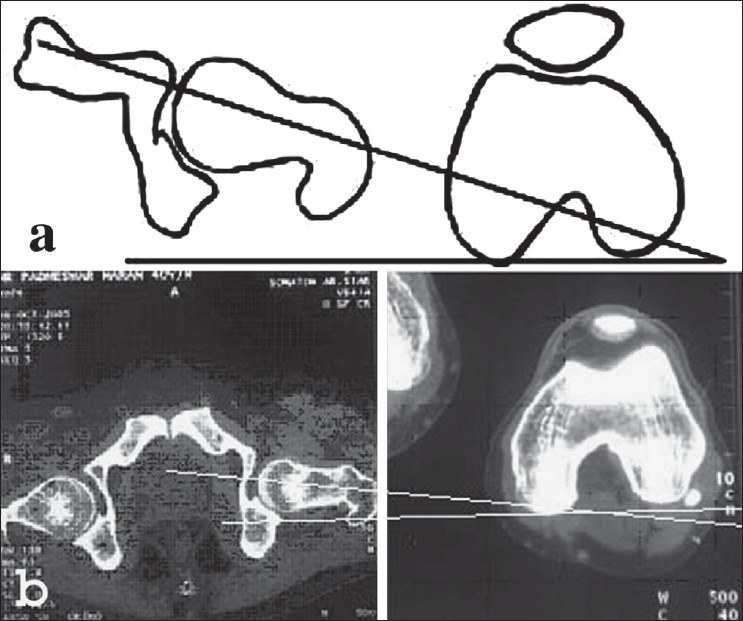
Line diagram (a) and CT scan (b) of femoral neck showing decreased femoral anteversion (8 degree)

**Figure 6:**
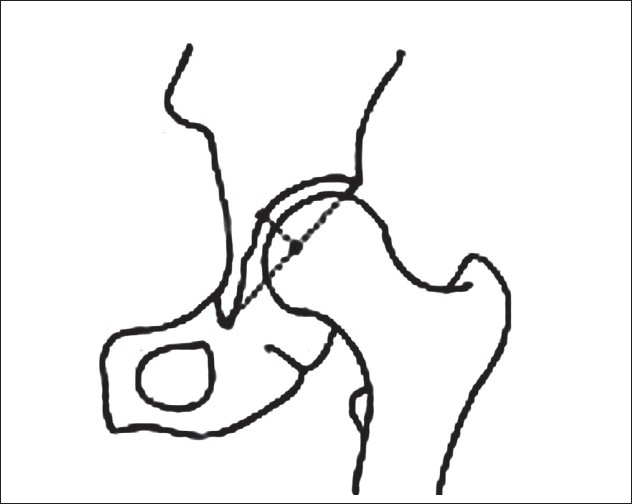
Line diagram of acetabular depth

**Figure 7:**
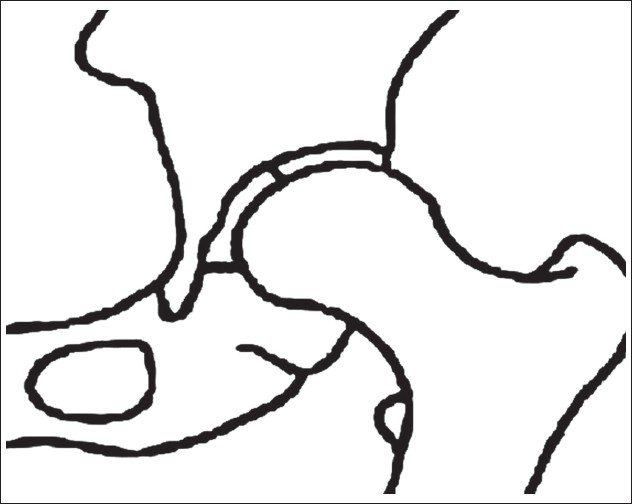
Line diagram of joint space width

Definition of parameters used are as follows: (i) CE angle of Wiberg; the angle between a line drawn vertically through the center of the femoral head and a second line drawn from the center of the femoral head to the anterior edge of the acetabulum^6^ [Figure 1]. (ii) Acetabular angle of Sharp; the angle between the horizontal line drawn through the tip of pelvic tear drop and a line from the tip of the tear drop to the anterior edge of the acetabulum^9^ [Figure 2a-c]. (iii)Neck shaft angle; the angle between the femoral shaft axis and the femoral neck axis^10^,^11^ [Figure 3a,b]. The femoral shaft axis is determined by a line drawn through the center of the medullary canal along the axis of the femur. The neck axis is drawn in the center of the femoral neck by joining two points equidistant from the superior and inferior surface of the femoral neck and parallel to the neck of the femur. (iv) Acetabular version; the angle between a perpendicular drawn to the line connecting the posterior ischia and a line connecting the posterior and anterior margins of the acetabulum^12^,^13^ [Figure 4a,b]. (v) Femoral Neck anteversion; the angle between the femoral neck axis and the condylar axis^14^,^15^ [Figure 5a,b]. The neck axis was drawn as explained previously in this section. Condylar axis is drawn by joining the two most posterior aspects of the femoral condyles. (vi) Acetabular depth; the distance from the center of a line connecting the anterior and the inferior acetabular margins up to the dome of the acetabulum^16^,^17^ [Figure 6]. (vii) Joint space width; we measured the joint space at three different levels [Figure 7]: (a) Superior edge of the acetabulum, (b) at the fovea, (c) inferior edge of the acetabulum.^2^ We also studied the vertical diameter of the femoral head and correlated it with the stature of the individuals. Vertical diameter was measured by the distance between the superiormost and inferiormost point of the femoral head taken perpendicular to the long axis of the femoral neck. All values were recorded on a Microsoft excel spreadsheet. We analyzed the variations between both the sexes and the age groups. Statistical analysis was done using Instat 3.5 software.

## RESULTS

We divided our study into age groups of 20-30 years (*n* = 20), 31-40 years (*n* = 27), 41-50 years (*n* = 25), 51-60 years (*n* = 12), 61-70 years (*n* = 8) and calculated the mean and standard deviation of the parameters in each group. The mean height of a Caucasian male was 165.6 cm (range 155.08-175.4), females 157.2 cm (range 145.4-170.32) and in the Mongoloids the mean height of males was 160.2 cm (range 150-170.32), females 152.6 cm (140.32-165.25) Observations in the different age groups are listed in Table 1. In both Mongoloids and Caucasoids the age group of 61-70 years showed extremes of the parameters, the highest was observed in the acetabular angle (mean = 39°, range = 36°-50°), neck shaft angle (mean = 140.2°, range = 134°-150°) and femoral neck anteversion (mean = 20.2°, range = 16°-45°), whereas the lowest was seen in the mean of CE angle (mean = 27.2°, range = 22°-60°), acetabular depth (mean = 1.7 cm, range = 1.5-5.6 cm) and joint space width (mean = 3.8 mm, range = 2.4-12 mm). The findings in the other age groups were inconsistent. We calculated the mean of males (*n* = 56) and females (*n* = 36) separately, there was no statistically significant difference in the mean of the parameters except for the femoral neck version which showed a 2° difference (*P* = 0.01), Table 2 shows the analysis of the parameters in males and females. Comparison was also made between the right and left sides of the individuals, in most of the parameters the values on the left side were higher, but they were statistically significant in the neck shaft angle: Right 139° (118°-142°); Left 140.7° (120°-150°) (*P*-value < 0.0001). The comparison of the left and right side is shown in Table 3.

**Table 1:** Influence of age on the different parameters in Mongoloids and Caucasoids (92 individuals)

NOS	Age (years) result	Acetabular angle (deg.)	CE angle(deg.)	Neck shaft angle (deg.)	Acetabular version (deg.)	Neck ante version (deg.)	Acetabular depth (cm)	Joint space width (mm)	Vertical diameter of femoral head (mm)
N = 20 (C = 12, M = 8)	20-30								
	T	39	33.2	139	18.4	18.2	2.6	4.8	41.2
	C	39.3	34	139.7	18.5	18.6	2.8	4.9	41.6
	M	38.7	32.4	138.3	18	17.8	2.4	4.7	40.8
	SD	4.1	8	6.9	5.2	4.2	0.9	2.2	2.4
N = 27 (C = 14, M = 13)	31-40								
	T	35.4	34.6	138	18.6	21.6	2.6	5.6	42.6
	C	36.2	34.9	138.8	18.8	21.7	2.7	5.8	43
	M	34.6	34.3	137.2	18.4	21.5	2.5	5.4	42.2
	SD	3	8.1	8.5	5.4	10.3	0.6	2.6	2.8
N = 25 (C = 13, M = 12)	41-50								
	T	36	35.1	140	16	20.5	2.2	5	43.5
	C	36.8	35.8	140.4	16.8	20.7	2.4	5.2	43.8
	M	35.8	34.4	139.6	15.2	20.3	2	4.8	43.2
SD	3.8	5.4	6.5	5.7	5.3	0.9	1.3	3.2
N = 12 (C = 5, M = 7)	51-60								
	T	38	28.4	139.1	20	21.2	2.4	4	44
	C	39.2	29	139.6	21	21.5	2.5	4.3	44.9
	M	36.8	27.8	138.9	19	20.9	2.3	3.7	44.1
	SD	4	6.1	8	5	2.8	0.6	0.5	2.6
N = 8 (C = 3, M = 5)	61-70								
	T	3.9	27.2	140.2	20.4	20.2	1.7	3.8	.6
	C	39.6	27.8	140.6	20.8	20.4	2	4	45
	M	38.4	26.6	139.8	20	20	1.4	3.6	44.2
	SD	4.2	3.2	5	5.5	4.6	0.5	0	2.8

SD - Standard deviation, deg. - degree, cm - centimeter, mm - millimeter; N - Number; T - total mean, C - Caucasian; M - Mongoloid; NOS - number of subjects

**Table 2:** Comparison between sexes

NOS		Acetabular angle (deg.)	CE angle (deg.)	Neck shaft angle (deg.)	Acetabular version (deg.)	Neck version (deg.)	Acetabular depth (cm)	Joint space width (mm)
56	Male							
	Mean	39	32.3	140	18	18	2.5	4.6
	SD	5.6	10.5	7.5	6.1	8	0.8	2
36	Female							
	Mean	39.4	33.5	13.9	18.4	20	2.5	4.4
	SD	3.2	10.2	7.1	6.2	7.5	0.6	2.1

SD - Standard deviation, deg. - degree, cm - centimeter, mm - millimeter; NOS - number of subjects

**Table 3:** Comparison between right and left sides

	Acetabular angle (deg.)		CE angle (deg.)		Neck shaft angle (deg.)		Acetabular version (deg.)		Femoral neck anteversion (deg.)		Acetabular depth (cm)		Joint space width (mm)	
							
	RT	LT	RT	LT	RT	LT	RT	LT	RT	LT	RT	LT	RT	LT
Mean	38.4	39.6	32	32.8	138	140	18.8	18	20.4	20.28	2.5	2.5	4.9	5
SD	5.19	5.4	10	10.6	7.24	7.92	5.3	5.7	5.7	5.1	.7	.8	2.2	2.2
Minimum	30	30	20	20	118	120	8	8	8	8	1.4	1.4	2	2
Maximum	50	50	60	60	142	150	40	40	45	40	5.6	5.6	12	12
Median	40	40	30	30	140	140	20	20	22	20	2.4	2.4	4	4

SD - Standard deviation, deg. - degree, cm - centimeter, mm - millimeter

The vertical head diameter was measured in all individuals on both sides. In Caucasians males and females mean was 44.6 mm (39-50 mm) and 42.3 mm (38-48 mm), respectively. In Mongoloid males and females mean was 43.05 mm (38-47 mm) and 40.75 mm (35.1-45.2 mm), respectively. The mean of the right and left side were also measured separately. The mean in Caucasian males: right was 44.3 mm (39-48 mm) and left was 44.9(40-50 mm). In Caucasian females the mean of right was 42.1 mm (38-47 mm) and of left was 42.5 mm (38-48 mm). In Mongoloid males: Right 42.8 mm (38-45 mm), left 43.3 mm (39-47 mm); in Mongoloid females: Right 40 mm (35-44 mm); left 41.5 mm (36-45.2 mm).

Overall results of the tomographic measurements in 92 individuals are listed in Table 4.

**Table 4:** Results of tomographic measurement showing mean, standard deviation, median and range (92 individuals)

	Mean	SD	Median	Range
Acetabular angle (deg)	39.2	4.9	40	30-50
CE angle of Wiberg (deg)	32.7	8.9	35	20-60
Neck shaft angle (deg)	139.5	7.5	140	118-150
Acetabular version (deg)	18.2	5.6	20	8-40
Femoral neck version (deg)	20.4	8.6	20	8-45
Acetabular depth (cm)	2.5	0.8	2.4	1.4-5.6
Joint space width (mm)	4.5	2	4	2-12

SD - Standard deviation, deg. - degree, cm - centimeter, mm - millimeter

## DISCUSSION

Differences in the parameters of bone and anatomical variations of the hip joint do exist among different races. The development of computed tomography has helped in further detailed anatomic study of the hip Joint. Tomographic study of pediatric hip has been reported mainly for detection of dislocation/subluxation or when measurement of femoral torsion and acetabular anteversion are needed^12^. We used CT scan to make a quantitative analysis of all the parameters of adult hip joint in our study. Our method is a collection of various parameters and their methods of measurement described by several authors.^2^,^3^,^8^–^11^,^13^–^17^

The CE angle was first described by Wiberg (1939)^6^ and subsequently by many authors.^6^,^10^,^11^ Values of >25° are considered normal whereas values < 20° are considered dysplastic. The CE angle of Wiberg studied in an adult Indian population by Mandal *et al*.^18^ found that in 83% the CE angle was between 28° and 42°. None of the hips had a CE angle of less than 20°, a similar pattern was seen when compared with Africans and Caucasians. Osteoarthritis is rare in Africans and Indians and fairly common in Caucasians. The finding of similar acetabular measurements in adult hips in these three races suggests that acetabular dysplasia may not have a significant role in the development of osteoarthritis.^18^ In our series the mean CE angle is 32.7° (range = 20°-60°; SD 8.9°). The CE angle was significantly higher (*P* = 0.002) in Caucasoids than the mongoloids, however, we are unable to correlate whether this higher value of CE angle in Caucasoid has an influence in the predisposition to osteoarthritis, as no study on the prevalence of osteoarthritis among the ethnic groups of NE region is currently available.

The acetabular angle was first described by Sharp.^9^ Acetabular angle is frequently used to determine the presence of dysplasia, values of >43° are considered dysplastic. Stuberg and Harris reported a mean acetabular angle of 32.2° in white males and 32.1° in white females respectively.^19^ Nakamura *et al*. reported a mean of 38° and a standard deviation of 3.6° in the Japanese population.^20^ In the present series we have found a mean acetabular angle of 39.2° (range = 30°-50°; SD 4.9°).

The femoral neck shaft angle has been examined by several authors and most authors agree that there is considerable individual variation and wide standard deviation in this angle. Hoaglund and Low stated that the average neck shaft angle in adults is 135°.^21^ Lequesne *et al*. found a standard deviation of 4.37°, the mean value was 132.8° in their study.^2^ In our series the mean was 139.5° (range = 118°-150°; SD 7.5°) and was several degrees more than the others. The neck shaft angle showed the highest variation when compared with the western literature and also between the Mongoloids and Caucasoids. Statistically significant variation was observed between the left and right side only (*P* = <0.0001).

The acetabular version was measured by Reikeras *et al*., they compared the measurements among normal and in osteoarthritic hip. The normal mean and standard deviation were 17°and 6°, respectively. They found no difference in mean of the acetabular angle in normal and osteoarthritic individuals.^22^ In the present study we have found an average of 18.2° (range = 8°-40°; SD 5.6°).

The femoral neck anteversion has been measured by various authors using plain radiograph/CT scan, clinically, as well as on dry specimens.^14^,^22^,^23^ The literature has suggested that the measurement on CT scan is more accurate than on X-rays.^14^,^23^ Hoaglund and Low^21^ in 1980 did a cadaveric study in Caucasians and Hong Kong Chinese, the results obtained were as follows. In Caucasians: Male 14° (4°-36°), Female 16° (7°-28°). In Hong Kong Chinese: males 14° (4°-36°) and 16° in females (7°-28°). Reikeras *et al*. measured the femoral neck anteversion (FNA) in normal and in osteoarthritic hip with CT scan and found it to be 13° with a standard deviation of 7° in the normal. In osteoarthritic hip they found an average of 6° more than the normal average.^22^ Jain *et al*. calculated femoral anteversion in dry specimens, as well as living persons using clinical, CT and biplane radiograph. They found CT to be accurate on living subjects, the mean FNA with the CT method was 7.4° (SD 4.6°).^23^ They also made a comparison between preoperative, clinical and biplane X-ray methods and found that the angle of anteversion of the neck of femur in humans exhibits a wide range (−25° to +50°) with the mean angle varying from 8° to 25°. They concluded that the hip joints of the Indian population would be evolutionally different from their Western counterparts, since our population is more apt to floor level activities with increased external rotation of the hip.^24^ Nagar *et al*. concluded from their study on adult Indian dry femora and normal subjects that average anteversion in male bones is greater and right-left variations exist, being greater on the right side.^25^ On clinical assessment they found a similar pattern. In our series the mean femoral neck anteversion was found to be 20.4° (range = 8°-45°; SD 8.6°). The mean in our series was more than most of the other series that we compared. Moderately decreased femoral neck anteversion (10°-14°) was observed in two hips.

The acetabular depth has been regarded by many authors as an important measurement to define acetabular dysplasia.^5^,^17^ An acetabular depth of less than 0.9 cm is considered dysplastic. We found a mean acetabular depth of 2.5 cm (range = 1.4-5.6 cm; SD 0.8 cm). Significant variation in the acetabular depth was observed among the Caucasoids and Mongoloids (*P* = 0.001); however, the mean values of acetabular depth was identical when comparison was made between right side: 2.5 cm (1.4-5.6 cm) and left side: 2.5 cm (1.4-5.6 cm), identical values were also observed between males: 2.5 cm (1.6-5.6 cm) and females: 2.5 cm (1.4-5.2 cm).

The joint space width measurement has been conducted by several authors to determine the normal mean and range. Most studies reveal a normal mean of about 4 mm.^10^ Joint space width is an important determinant of osteoarthritic changes. Many authors have concluded that joint space narrowing should not be expected in an elderly or obese person unless arthritic changes develop.^2^,^16^ The average joint space in our study was 4.5 mm (range = 2-12 mm; SD 2 mm). We observed no significant change among the different age groups.

Parameters measured by various authors concluded that the value was higher on the left side than on the right side.^26^,^27^ We have also found a higher value on the left side in most of the parameters but it was statistically significant in the neck shaft angle (120°-150°) only (*P* < 0.0001).

Several authors have suggested that the vertical diameter of the femoral head is larger in taller individuals.^17^,^28^ A study conducted on the femora of Nigerians revealed a mean diameter of 54.23 in males and 54.08 in females.^28^ Chauhan *et al*. reported a mean vertical diameter of femoral head in males and females to be 45.44 mm and 43.87 mm, respectively on the right side and 45.84 mm and 44.67 mm, respectively on the left side.^17^ We also agree with the reported conclusions, since the mean vertical diameter of the femoral head in our individual is less that of the Nigerians and the North Indians as the average height of the people of the northeast are shorter, this was also obvious when we compared the mean vertical diameter between the Caucasians and the Mongoloids of our own subjects, Caucasians are taller than Mongoloids and are expected to have larger diameter of femoral head.

All the parameters were found to be in the higher range in the Caucasoids than the Mongoloids, but statistically significant variation was found in the acetabular depth, (*P* = 0.001) and CE angle of Wiberg (*P* = 0.002) apart from the vertical diameter of the femoral head (*P* = 0.01). The increased acetabular depth and CE angle of Wiberg in the Caucasians is due to a larger head diameter.

There was variation in the parameters among the age groups that we measured, but no regular pattern of increase or decrease in relation to the age group was observed. The age group of 61-70 years showed the highest value in the acetabular angle, neck shaft angle and femoral neck anteversion, the same age group showed the lowest value in CE angle, acetabular depth and joint space width. The authors could not find any valid explanation for the variations in the age groups, measurement was more towards the extremes.

The Indian subcontinent comprises a vast collection with different morphological, genetic, cultural and linguistic characteristics, while much of this variability is indigenous, a considerable fraction of it has been introduced through large-scale immigration into India in historical times.

The northeastern part of India is inhabited by numerous endogenous tribes and castes that have their own distinct social, linguistic and biological identity. It has been hypothesized that a plethora of migration, particularly through the northeast Indian corridor has contributed to the present day population of northeastern India. Ethnically speaking, most of the tribal groups are Mongoloids; whereas caste groups are either Caucasoids or show a mosaic of features of both the ethnic groups. These factors are responsible for wide variations of different parameters in the individuals of the NE region.

Both the Mongoloids and Caucasoids show a certain degree of differentiation within themselves in work culture, habits and biological traits such as anthropometry, genetic markers and dermatoglyphics.^29^

Knowledge of the anatomical parameters of the bony components of the hip joint is very essential, as it will help better understanding of the etiopathogenesis of diseases like primary osteoarthritis of the hip joint. Awareness of the average dimensions of the acetabulum and femoral head will assist prosthetists in designing a suitable prosthesis according to the need of a particular individual.^17^,^23^ The parameter and its values in our series shows differences when compared to the other western literature. The limitation of this study has been a small sample size hence a study with a larger sample size is warranted.
